# Heterotopic Bone Formation in Juvenile Polyp: A Histopathological Entity

**DOI:** 10.14309/crj.0000000000000705

**Published:** 2022-01-11

**Authors:** Madhusmita Mohanty, Neha Pandey, Urmila Senapati, Preetam Nath

**Affiliations:** 1 Department of Pathology, Kalinga Institute of Medical Sciences, Bhubaneswar, India; 2 Department of Gastroenterology, Kalinga Institute of Medical Sciences, Bhubaneswar, India

## CASE REPORT

A 5-year-old boy presented with a 1-month history of blood in stools. The blood was bright red in color, moderate in amount, and associated with intermittent abdominal pain. He had no history of pain during defecation, no constipation, and no alteration in frequency or consistency of stool. Systemic examination revealed only pallor. Local examination of the boy was unremarkable.

**Figure 1.**
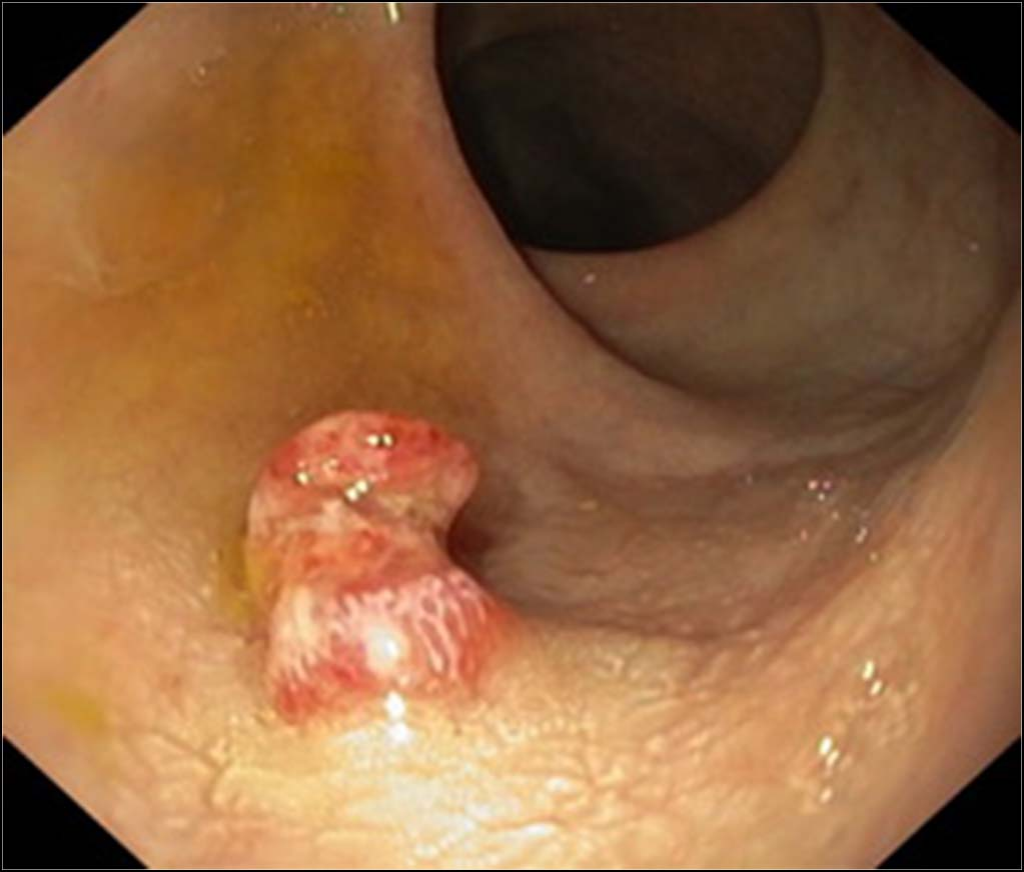
Colonoscopy showing polyp in the rectum.

Colonoscopy examination revealed terminal ileitis with a rectal polyp (Figure 1). A polypectomy was performed, and a specimen was sent for histopathological examination. Grossly, a polypoidal tissue cut section measuring 1.0 × 0.5 × 0.3 cm was firm and greyish white. Microscopic examination showed a polyp with mucosal surface ulceration. Numerous cystically dilated and tortuous crypts filled with inspissated mucin were seen. Intervening lamina propria was edematous and filled with mixed inflammatory cells. One focus showed bony trabeculae rimmed by osteoblast. We diagnosed our patient with juvenile polyp with osseous metaplasia.

**Figure 2.**
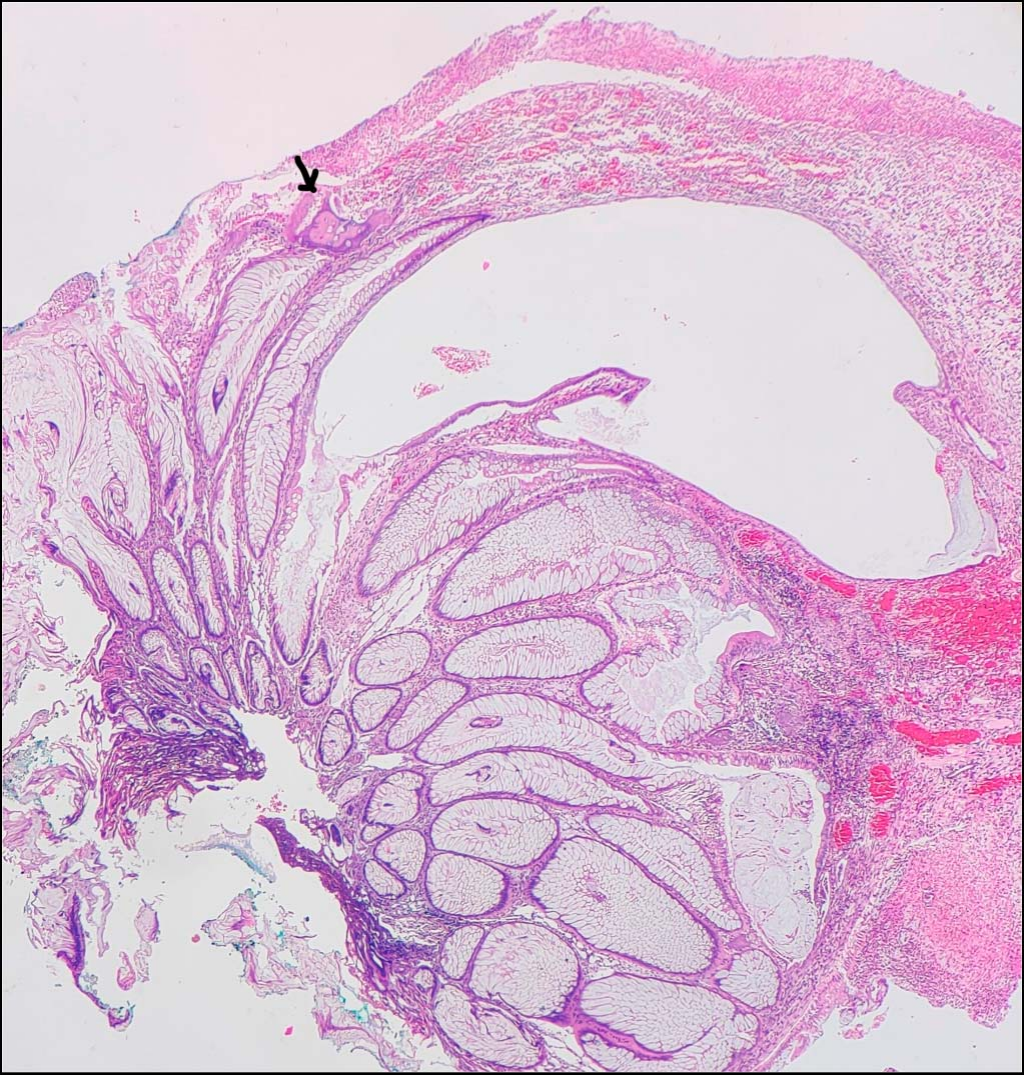
Hematoxylin-eosin stain, ×40 magnification. Juvenile polyp having surface ulceration, and a single black arrow showing osseous metaplasia.

**Figure 3.**
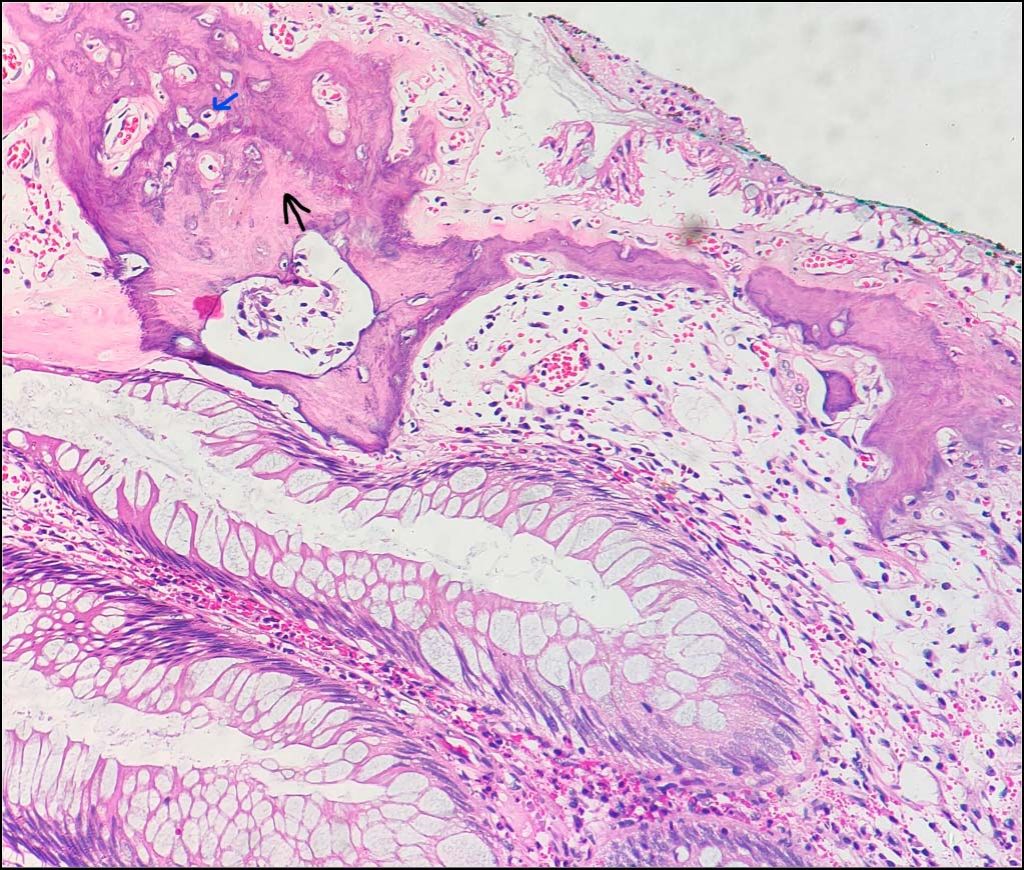
Hematoxylin-eosin stain, ×200 magnification. Juvenile polyp, a single black arrow showing bony matrix, and a single blue arrow showing osteocytes in lacunae.

Juvenile polyps are hamartomatous polyps which may undergo osseous metaplasia. In most recent studies, expression of bone (Figures 2 and 3) morphogenic proteins had been observed in the setting of osseous metaplasia.^1^ Osseous metaplasia, ie, heterotopic bone formation, is an adaptive response to any chronic inflammation. Only a few cases of neoplastic and nonneoplastic gastrointestinal polyps are associated with this extremely rare type of metaplasia.^2^ It is most commonly seen in the colorectal region in association with epithelial polyps. To the best of our knowledge, only 9 other cases of osseous metaplasia in juvenile polyps have been reported so far worldwide.^2^

## DISCLOSURES

Author contributions: M. Mohanty edited the article, reviewed the literature, provided images, and is the article guarantor. N. Pandey wrote the article and reviewed the literature. U. Senapati revised and approved final the article. P. Nath provided images and revised and approved the final article.

Acknowledgments: We thank technician Niskara Sahoo for their contribution in good section cutting.

Financial disclosure: None to report.

Informed consent was obtained for this case report.
